# Non-Celiac Gluten Sensitivity: An Update

**DOI:** 10.3390/medicina57060526

**Published:** 2021-05-24

**Authors:** Feliznando Isidro Cárdenas-Torres, Francisco Cabrera-Chávez, Oscar Gerardo Figueroa-Salcido, Noé Ontiveros

**Affiliations:** 1 Doctorate Program in Nutrition Science, Faculty of Nutrition Sciences, University of Sinaloa, Culiacán 80019, Mexico; feliznando@uas.edu.mx (F.I.C.-T.); fcabrera@uas.edu.mx (F.C.-C.); 2 Postgraduate in Health Sciences, Division of Biological and Health Sciences, University of Sonora, Hermosillo 83000, Mexico; 3 Clinical and Research Laboratory (LACIUS, URS), Department of Chemical, Biological, and Agricultural Sciences (DC-QB), Division of Sciences and Engineering, University of Sonora, Navojoa 85880, Mexico

**Keywords:** gluten, NCGS, wheat sensitivity, NCWS

## Abstract

Non-celiac gluten sensitivity (NCGS) is a clinical entity characterized by the absence of celiac disease and wheat allergy in patients that trigger reproducible symptomatic responses to gluten-containing foods consumption. Due to the lack of sensitive and reproducible biomarkers for NCGS diagnosis, placebo-controlled gluten challenges must be carried out for its diagnosis. The gluten challenges can be either double- or single-blind, for research or clinical practice purposes, respectively. For improving our understanding about the magnitude and relevance of NCGS in different populations, epidemiological studies based on self-report have been carried out. However, the gluten challenge-based prevalence of NCGS remains to be estimated. Since NCGS was recently recognized as a clinical entity, more studies are needed to delve into NCGS pathogenesis, for instance, the molecular interactions between the suspected cereal grain components that trigger NCGS, such as fermentable oligo-, di-, monosaccharides, and polyols (FODMAPs) and amylase and trypsin inhibitors, and the immune system remains to be elucidated. Although still under debate, NCGS patients can be susceptible to only one or more than one of the NCGS triggers. The treatment of NCGS involves the dietary restriction of the suspected triggers of the disease, but there is controversial data about the effectiveness of different dietary interventions such as the gluten-free diet and low-FODMAP diet. Certainly, our understanding of NCGS is improving quickly due to the constant availability of new scientific information on this topic. Thus, the aim of the present narrative review is to present an up-to-date overview on NCGS from epidemiology to current therapy.

## 1. Introduction

Gluten-containing grains have become a fundamental part of the human diet being wheat the most consumed cereal around the world [1]. Wheat is widely utilized in the food industry and nutritionally contributes to the human diet [2,3]. However, some disorders may occur when consuming some specific wheat components, such as gliadins, glutenins, and fermentable oligo-, di-, monosaccharides, and polyols (FODMAPs). These disorders are known as gluten-related disorders (GRDs) and mainly involve celiac disease (CD), wheat allergy (WA), and non-celiac gluten sensitivity (NCGS) [4]. CD is an enteropathy with autoimmune characteristics and it is triggered by gluten-containing foods in susceptible individuals that carry human leukocyte antigen (HLA)-DQ2 and/or HLA-DQ8 haplotypes [5]. WA is characterized by the production of IgE antibodies against wheat proteins and the development of symptoms of immediate-type food allergy [6]. NCGS is characterized by the triggering of intestinal and/or extraintestinal symptoms after the consumption of products made with gluten-containing cereals, but both CD and WA must be properly ruled out as the symptoms overlap among the clinical entities and there is a lack of sensible and specific biomarkers for NCGS diagnosis [7]. The first insights of NCGS were published more than 40 years ago [8,9]. However, the triggering of wheat-induced symptoms in subjects that underwent double-blind placebo-controlled (DBPC) gluten challenges and in whom CD and WA were ruled out was reported until the beginning of the second decade of the 21st century [10,11]. These reports gave rise to intensive research to elucidate the underlying mechanisms of NCGS, identify the specific triggers of the disease and biomarkers for its diagnosis, and know the best treatment of NCGS and its epidemiology. Certainly, research on NCGS is still increasing and new information on this topic is published every day. Thus, the aim of this narrative review is to present an up-to-date overview on NCGS considering the fundamental factors of this clinical entity and covering different topics, from epidemiology to current therapy.

## 2. Definition

NCGS is defined as “a syndrome characterized by intestinal and extra-intestinal symptoms related to the ingestion of gluten-containing food, in subjects that are not affected by either CD or WA” [12]. The exclusion of CD and WA for the diagnosis work-up of NCGS remains as a key step due to the lack of biomarkers for NCGS diagnosis. Importantly, NCGS cases do not develop intestinal damage or sensitization to wheat proteins as it happens in CD and WA, respectively. The genetic background that underlies NCGS is uncertain although compelling evidence highlights that the CD predisposing haplotypes HLA-DQ2 and/or HLA-DQ8 have no relevance for triggering the condition. Evidence also suggests that wheat components other than gluten, such as FODMAPs, and amylase and trypsin inhibitors (ATIs), could also act as triggers of some clinical manifestations in NCGS cases, either intestinal or extraintestinal or both [13,14]. Alternatively, the term “non-celiac wheat sensitivity” has been proposed instead of NCGS, as the word wheat encompasses all the components involved in the grain [15]. Main limitation of the term “non-celiac wheat sensitivity” is that exclude other grains, such as rye, barley, and oats, that may contain the components that trigger the symptoms. Thus, there is still a need for a consensus on a term that properly encompasses the triggers of NCGS and perhaps define the syndrome.

## 3. Epidemiology

The prevalence of NCGS remains unknown in many regions around the world. The lack of NCGS epidemiological studies in many populations can be mainly attributed to the recent recognition of the disease by the scientific community and the lack of sensitive and reproducible biomarkers for its diagnosis [16]. Additionally, the absence of an adequate diagnosis approach to be used in clinical practice complicates the identification of NCGS cases [17]. Consequently, NCGS epidemiological studies carried out at population level are survey-based cross-sectional ones that estimate the self-reported prevalence of NCGS, either through face-to-face interviews or using online platforms [18]. In general, the identification of self-reported NCGS cases in survey studies is based on the following criteria: (1) Self-reported adverse reactions to wheat/gluten; (2) absence of self-reported physician diagnosis of CD and/or WA and (3) adherence to a gluten-free diet (GFD). Data obtained from survey studies suggest that the prevalence rates of NCGS range from 0.49% to 14.9%, which are higher than almost all the prevalence rates estimations of CD or WA [19,20,21,22,23,24,25,26,27,28,29,30,31]. Survey-based cross-sectional studies have the limitation that their results are not corroborated with objective diagnostic tests (i.e., HLA typing, serological tests, oral challenges) to rule out CD or WA. These studies generate valuable information at population level which could serve as groundwork for further epidemiological studies based on objective diagnostic criteria, but the prevalence estimations should be interpreted with caution. The high heterogeneity in the prevalence rates of self-reported NCGS can be mainly attributed to the use of different instruments (i.e., questionnaires), the target population, and the approaches used for data collection (Table 1). Special attention should be paid to the criteria utilized to define self-reported NCGS cases, as these criteria directly impact on the prevalence rates estimations. Additionally, the socioeconomic index, dietary, and cultural aspects could also affect the prevalence rates estimations of NCGS. In fact, it has been suggested that the per capita consumption of wheat among the populations is related to the prevalence rates of GRDs (increased consumption of wheat equals to an increased prevalence rates of GRDs), but further studies are needed to establish a direct relationship between wheat consumption and NCGS prevalence rates. Survey studies that estimate the prevalence rates of NCGS in the Latin American region utilized the same instrument in all the countries surveyed. These studies reported that the prevalence of NCGS ranges from 0.49% to 6.28% in the general adult population [24,25,26,27,28,29]. Other studies carried out in Australia have reported NCGS prevalence rates as high as 14.9% in adult population, but both NCGS criteria and the instrument utilized were different than the ones utilized in Latin America [20,21]. These facts highlight that consensus is needed to define the criteria to identify potential NCGS cases under the bases of a self-report approach.

## 4. Clinical Picture

**Figure 1:**
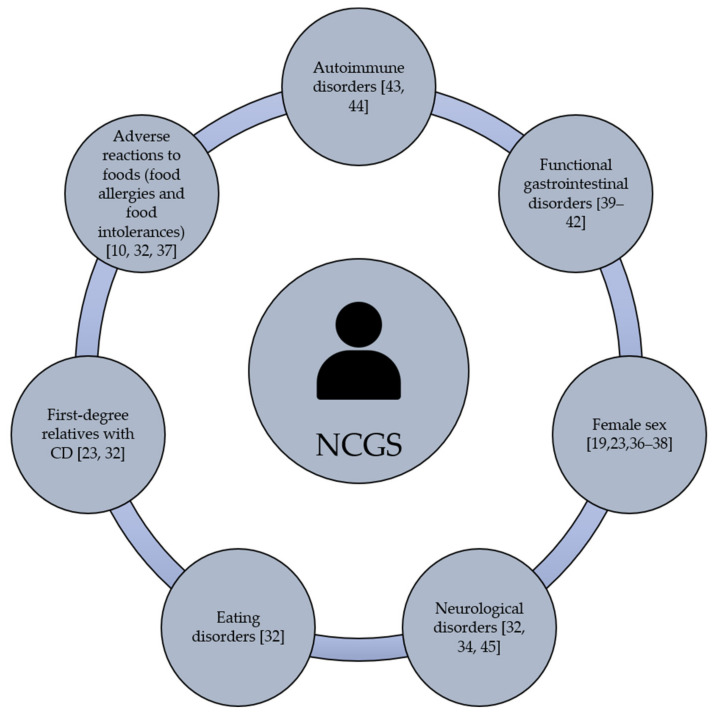
Potential predisposing factors of NCGS.

Gastrointestinal and/or extraintestinal symptoms can be triggered in NCGS cases. The most common gastrointestinal symptoms are bloating, abdominal pain, diarrhea, nausea and reflux [32,33]. The extraintestinal manifestations are widely heterogeneous and include headache, general tiredness, blurred mind, fibromyalgia, lack of well-being, dermatitis, joint pain and depression [34]. Based on self-reported NCGS cases, gastrointestinal symptoms are more common than extraintestinal ones [21,22,26,27,35]. Once the grain components that trigger NCGS were consumed, the clinical manifestations of the condition can appear after hours or days [12]. Current evidence is not enough to establish the individual predisposition to NCGS, although this condition could be more prevalent in females than males, particularly in young to middle-adults [19,23,36,37,38]. Beyond gender, it remains under debate if NCGS is more prevalent in subjects with clinical history of autoimmune and functional gastrointestinal disorders [39,40,41,42]. In fact, autoimmune diseases are present in 24% to 25.3% of subjects with a well-defined diagnosis of NCGS being autoimmune thyroiditis the most common autoimmune disease (69.5% to 100%) [43,44]. Others have shown that NCGS is associated with microscopic enteritis (11 out of 22 patients, 50%), neurological disorders, eating disorders, adverse reactions to foods (i.e., food allergies and food intolerances), the presence of anti-nucleus antibodies, and having first-degree relatives with CD, [20,23,27,45] (Figure 1). All these potential predisposing factors have been documented, but much remains to be done regarding the molecular mechanisms or genetic bases that link NCGS with other disorders.

## 5. Current Knowledge on the Pathogenesis of NCGS

Although the pathogenesis of NCGS remains unknown, data show that there is a predominant role of the innate immune system. Increased expression of toll-like receptor (TLR) 2 and decreased expression of the T-regulatory cell marker factor forkhead box P3 (FOXP3) can be found in intestinal biopsies from NCGS patients in comparison to healthy subjects and CD patients [40]. Up-regulated levels of interleukin (IL) 10, transforming growth factor (TGF) α, C-X-C motif chemokine ligand 10 (CXCL-10), and granulocyte-macrophage colony-stimulating factor (GM-CSF), CD14, and lipopolysaccharide (LPS)-binding protein have also been reported in wheat-sensitive individuals in the absence of CD [46,47,48]. Furthermore, the expression of RNA transcripts that could be implicated in the activation of the innate immune system, such as zurocidin 1 (AZU1), bone morphogenetic protein-7 (BMP7), cluster of differentiation 70 (CD70), among others, have been documented in the intestinal mucosa of NCGS patients [49]. These data support the notion that there is a predominant role of the innate immune system in NCGS. However, evidence also suggest the involvement of the adaptive immune system, as there is an increase of anti-gliadin antibodies (AGA) in approximately 50% of NCGS patients [50]. Regarding interferon gamma (IFN-γ), increased levels of this cytokine has been found in the intestinal mucosa of NCGS patients after a gluten challenge [51,52], but the role of IFN-γ in NCGS has been questioned [53,54].

Several studies have reported that, at intestinal level, changes occur in NCGS patients. Particularly, intestinal inflammation can be relevant in the pathogenesis of NCGS. Increased levels of eosinophils, intraepithelial CD3^+^ T cells, and lamina propria CD45^+^ cells have been reported in duodenal and rectal tissues from NCGS patients [37]. Although NCGS patients do not show an altered villous architecture as seen in CD cases, a high percentage of NCGS patients presents a slight increase of intraepithelial lymphocytes (IELs) [37,38]. Increased levels of mast cells in the duodenum have also been reported [55], and this correlates with a higher intensity of abdominal pain and bloating in NCGS individuals [56]. On the other hand, the production of tumor necrosis factor (TNF) α by CD45^+^, CD3^+^, CD4^+^, and CD8^+^ cells and IL-17 by CD4^+^ cells is higher in the rectal tissue of active NCGS patients than in healthy controls, suggesting that the adaptive immune system is involve in the pathogenesis of NCGS [57]. Others have reported an increased percentage of cells that express cytokines that induce and maintain Th1 and Th17 responses, such as IL-12, IL-15, and IL-2, and cells that express TNF-α and IL-1β suggesting a concomitant role of both the innate and adaptive immune system in NCGS [58]. Therefore, evidence suggests that both the innate and adaptive immune systems trigger the intestinal inflammation that occurs in NCGS cases.

An intestinal barrier dysfunction has been suggested to play a role in the pathogenesis of NCGS. A study by Uhde et al. reported that NCGS individuals present increased serum levels of soluble CD14, lipopolysaccharide (LPS)-binding protein, and antibody reactivity to microbial products (LPS, flagellin). These biomarkers correlate with the serum levels of intestinal fatty acid-binding protein (FABP2), a biomarker for the detection of in intestinal injury. The translocation of microbial products due to an altered intestinal barrier function could contribute to the activation of the innate and adaptive immune systems, triggering a systemic immune response [59]. Other findings also suggest an intestinal barrier dysfunction in NCGS patients due to the transepithelial electrical resistance in intestinal explants from NCGS patients is decreased in comparison to patients with CD in remission [60]. Additionally, an intestinal dysbiosis has also been reported in NCGS patients, and some authors suggest that this could contribute to the intestinal barrier dysfunction [61]. Overall, current evidence suggests that the pathogenesis of NCGS involve changes at intestinal level (inflammation, dysbiosis, and altered barrier function), the translocation of microbial and dietary products, and activation of the innate and adaptive immune systems.

**Figure 2:**
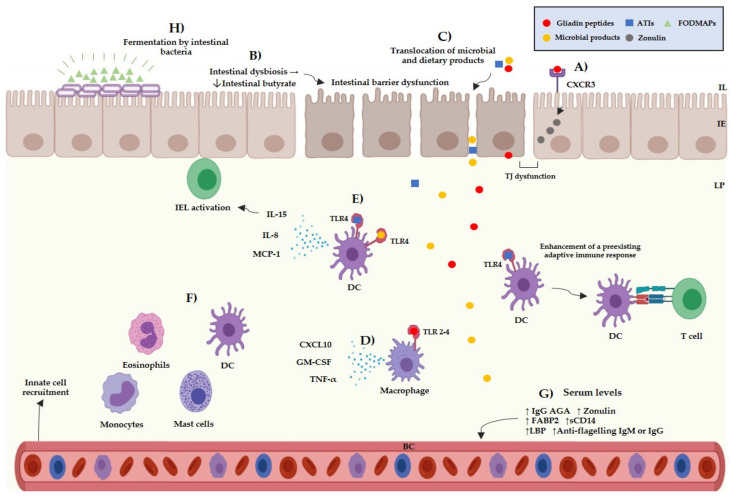
Current evidence of the potential pathogenic mechanism of NCGS. (**A**) Interactions between gliadin peptides and CXCR3 receptors in the intestinal epithelium trigger the release of zonulin increasing the intestinal permeability. (**B**) A chain of reactions that involves the decrease of intestinal butyrate, increased levels of FABP2 and low levels of intestinal alkaline phosphatase is induced by an intestinal dysbiosis, which can induce an intestinal barrier dysfunction. (**C**) Microbial and dietary products can reach the lamina propria from the intestinal lumen due to an increased intestinal permeability. (**D**) Interactions between gliadin peptides and toll-like receptors 2–4 can occur triggering the release pro-inflammatory cytokines, such as CXCL10, GM-CSF, and TNF-α by myeloid cells. (**E**) Interactions between the TLR4-MD2–CD14 complex and ATIs/LPS could trigger the release of pro-inflammatory cytokines, such as IL-8, MCP-1, and IL-15 by myeloid cells. ATIs can enhance the adaptive immune response in the gut associated lymphoid tissue inducing the antigen presentation to T cells. (**F**) The release of pro-inflammatory cytokines can promote the recruitment of myeloid cells in the lamina propria, such as mast cells, eosinophils, monocytes, and dendritic cells, triggering local inflammation. (**G**) Microbial products can reach the blood stream triggering a systemic immune response. (**H**) FODMAPs can be fermented by the intestinal bacteria, giving rise to intestinal luminal distention. IL: intestinal lumen, IE: intestinal epithelium, LP: lamina propria, BC: blood circulation, TJ: tight junction, ATIs: amylase and trypsin inhibitors, FODMAPs: fermentable oligo-, di-, monosaccharides, and polyols, IEL: intraepithelial lymphocytes, DC: dendritic cell, TNF-α: tumor necrosis factor α, MCP-1: monocyte chemoattractant protein-1, GM-CSF: granulocyte-macrophage colony-stimulating, AGA: anti-gliadin antibodies, FABP2: intestinal fatty acid-binding protein 2, LBS: lipopolysaccharide-binding protein.

Besides the multifactorial background of the NCGS pathogenesis, there are different subsets of patients, which can be sensitivity to different cereal components. The main components suspected to trigger symptoms in NCGS are gluten, ATIs, and FODMAPs, either individually or in combination [62]. Gluten is a complex of different hydrophobic proteins (gliadins: alcohol-soluble and glutenins: soluble in weak acids) and accounts for 80–85% of the total protein content of wheat [63]. The role of gluten as the main trigger in NCGS is questionable; for instance, a meta-analysis study reported that only 16% of NCGS patients informed gluten-specific symptoms [64]. Other studies reported that 36% of potential NCGS cases informed symptomatic relapse after undergoing a gluten challenge and that 31% informed symptomatic relapse after a placebo challenge [65]. Thus, the specific role of gluten in the pathogenesis of NCGS and as the trigger of NCGS related symptoms is still not fully understood. Gluten can interact with the intestinal epithelium through the C-X-C Motif Chemokine Receptor 3 (CXCR3) promoting the release of zonulin by enterocytes [66], and allowing the passage of molecules from the intestinal epithelium towards the lamina propria. Once gliadin peptides have entered the lamina propria, they could activate the innate immune system via TLR-2 and TLR-4 receptors, inducing the release of pro-inflammatory cytokines such as IP-10/CXCL10 and TNF-α (Figure 2) [67].

ATIs are a group of low-molecular proteins that are highly resistant to gastrointestinal proteases and can be found in the endosperm of plant seeds, where they act as natural pesticides. Although the role of ATIs in NCGS remains uncertain, they have been proposed as molecules with the potential to activate the innate immune system in NCGS [68]. Both in vitro and in vivo studies have reported that ATIs can activate the innate immune system through interaction with the toll like receptor 4–myeloid differentiation factor-2– cluster of differentiation 14 (TLR4–MD2–CD14) complex. This event induces the activation of nuclear factor kappa-B and the release of pro-inflammatory cytokines, such as IL-8, IL-15, TNF-α, and MCP-1 (Monocyte Chemoattractant Protein-1), by dendritic cells, macrophages, and monocytes [69,70] (Figure 2). An intestinal barrier dysfunction could allow ATIs to reach the lamina propria and interact with immune cells. Leccioli et al. stated the hypothesis that the pathogenic mechanism of NCGS may involve an intestinal dysbiosis characterized by a decrease of *Firmicutes* and/or *Bifidobacteria*, giving rise to low production of intestinal butyrate. In this context, a chain of events that involves low levels of intestinal alkaline phosphatase, intestinal damage, increased levels of FABP2, and the translocation of LPS and intact ATIs to the lamina propria could occur. Once there, LPS and ATIs could trigger the release of pro-inflammatory mediators leading to a local and systemic inflammation [71] (Figure 2). The previous hypotheses remain to be corroborated as no study has shown ATIs to have relevance triggering the symptoms reported by NCGS patients.

FODMAPs are a group of carbohydrates including fructose, lactose, glucose, polyols, fructans, and galacto-oligosaccharides [72]. FODMAPs could trigger symptoms in different gastrointestinal disorders, including NCGS [73]. Skodje et al. reported that 59 patients with self-reported NCGS presented a higher overall symptoms score after a fructan challenge than after a gluten or placebo challenge [13]. Additionally, NCGS patients have reported remission of symptoms after following a low-FODMAP diet [74]. FODMAPs can be fermented by intestinal bacteria giving rise to intestinal luminal distention due to an increase of luminal water content and gas production [75] (Figure 2). Luminal distention could cause the stimulation of intestinal mechanoreceptors and stimulate the enteric nervous system, giving rise to neuropsychiatric symptoms [76]. It is still difficult to know the specific role of FODMAPs and other cereal components in the development of NCGS. Therefore, researches should make efforts to establish the specific role of the dietary components that trigger the symptoms in NCGS patients [77].

## 6. Diagnosis

There is a lack of sensitive and specific biomarkers for the diagnosis of NCGS [16] and, consequently, its diagnosis is based on the exclusion of CD and WA and the clinical assessment of the patient while undergoing a double- or single-blind placebo-controlled gluten challenge. The Salerno experts ‘criteria established a standardized approach for the diagnosis of NCGS. This approach involves the clinical assessment of the patient while he/she is following a GFD for at least 6 weeks. After this period, a DBPC gluten challenge with a crossover approach should be performed (gluten and placebo challenge for one week each), but a single-blind placebo-controlled gluten challenge could be implemented in clinical practice [12]. However, this approach is difficult to apply in daily clinical practice due to most patients self-diagnose and start a GFD, and they are not willing to intake gluten again in most cases. Also, it is uncertain which are the main triggers of the symptoms, and it is possible the contribution of a nocebo response [76]. The German Society of Allergology and Clinical Immunology task force state that the current diagnosis of NCGS is inappropriate as there is a lack of validated diagnostic criteria, frequent self-diagnosis and self-instruction of a GFD among patients, the challenging identification of gluten as the main culprit, and numerous variables that complicates the clinical assessment of the patient during a GFD [17]. Despite the potential limitations, the Salerno expert’s criteria have shown to be useful for diagnosing NCGS, establishing a diagnose of NCGS in patients with self-reported NCGS with lower cut-off values compared to only a clinical diagnosis (63% and 85%, respectively) [78]. Additionally, the Salerno experts’ criteria have shown a direct relationship between the gluten challenge and the triggering of symptoms in 40% of self-reported NCGS patients [65].

Although the Salerno experts´ criteria establish the guidelines for the implementation of a DBPC gluten challenge, the methodological approaches used for carrying out the challenges for the diagnosis of NCGS do not adhere to the criteria. A meta-analysis that evaluated eleven studies that carried out gluten challenges under a DBPC basis reported that the type of vehicle used for the challenge, the amount of gluten, type of placebo, and the duration of the gluten/placebo challenge and washout period widely differ between the studies [65]. The heterogeneity found in DBPC studies for the diagnosis of NCGS complicates the comparison of the results across studies. In addition, it has been reported that 40% of patients exhibit a nocebo response, which could lead to an overestimation of the real prevalence of NCGS [64]. On the other hand, there is a lack of a standardized vehicle for performing the challenges for the diagnosis of NCGS. The lack of a standardized recipe for preparing such a vehicle further complicates the repeatability and comparison of the results across DBPC studies. Also, most studies do not report sensory evaluations to determine if the gluten vehicles and placebo used for carrying out the challenges are indistinguishable from each other, contributing to a possible increase in the nocebo response found in DBPC studies [79,80]. In this sense, researchers should make efforts for standardizing a methodology for developing such vehicles.

The absence of sensitive and specific biomarkers for NCGS diagnosis makes the identification of NCGS cases challenging. To date, numerous biomarkers have been suggested for the diagnosis of NCGS, such as the evaluation of eosinophils, intraepithelial CD3^+^ T cells, T helper lymphocytes, mast cells, cytokine and antibody serum levels, RNA transcripts and miRNA signatures [37,38,49,53,55,81,82,83] (Table 2). These biomarkers are not distinctive of NCGS, but their determination as clinical laboratory parameters allows the differentiation of potential NCGS cases from other diseases. Currently, there is a huge heterogeneity in the criteria used in clinical trials to define NCGS cases (patients with only self-report NCGS or with a well-defined NCGS diagnosis), which complicates fair comparisons of the results across studies. Additionally, the components used in clinical trials to evaluate the symptoms triggered in NCGS cases also differs (wheat as a whole, gluten, FODMAPs) [64,65]. Thus, the search for a diagnostic biomarker for NCGS is difficult, as there is a huge heterogeneity in the methodological approaches used in different studies.

### Overlapping with Other Diseases

As the clinical symptoms related to NCGS are widely diverse, they overlap with those found in other diseases, such as other GRDs (CD and WA) and functional gastrointestinal disorders, like irritable bowel syndrome (IBS) and functional dyspepsia [40,41,86]. The overlapping among symptoms of NCGS and other GRDs is common, NCGS cases could trigger intestinal and extraintestinal manifestations. However, patients with NCGS do not present IgA anti-TTG2 autoantibodies and specific IgE antibodies against wheat proteins [12,87]. Also, HLA DQ2/DQ8 haplotypes are only slightly associated with NCGS, approximately 50% of NCGS patients carry the haplotypes, in comparison to >95% in CD patients [35,61]. These biomarkers are necessary to properly distinguish NCGS from CD or WA cases. Furthermore, the symptoms triggered in NCGS are lower in intensity than those triggered in CD and WA and there is no evidence of long-term complications in comparison to CD [88,89]. The onset of the symptoms differs among GRDs, especially between WA and NCGS (minutes to hours and hours to days, respectively). These data could also be helpful in the overall clinical analysis to distinguish NCGS cases from CD and WA. In addition, patients with NCGS present a different subclass of IgG anti-gliadin antibodies in comparison with CD patients and healthy subjects (IgG4 and IgG2, respectively) [82]. On the other hand, although both NCGS and CD patients present a lesion type 1 according to the Marsh-Oberhuber classification (IELs >25/100 enterocytes), the distribution of the IELs in the intestinal epithelium of NCGS and CD patients differs [38,55]. In general, an in-depth analysis of the patients’ clinical history, the identification of wheat as the main trigger of the symptoms, the type, onset and intensity of the symptoms, and adequate knowledge of the protocols for diagnosing GRDs is necessary for correctly differentiating NCGS from CD and WA (Table 3).

IBS is one the most common gastrointestinal disorders with a global prevalence that ranges from 1.1% to 35.5% in the adult population [90]. Currently, IBS diagnosis is based on clinical assessment of patients using the Rome IV criteria [91]. IBS symptoms are commonly presented in NCGS (e.g., bloating, abdominal pain, changes in bowel habits) [42]. In fact, it has been reported that approximately 20% to 37% of patients with self-reported NCGS fulfill the criteria to be classified as IBS [19,31]. Due to the lack of sensitive biomarkers for the diagnosis of NCGS and IBS, their differentiation is complicated [92]. Current studies report that there is a cohort of IBS patients that are sensitive to wheat components, including gluten and FODMAPs [42,93]. Similar to NCGS, a GFD and a low-FODMAP diet can reduce the intensity of the symptoms in some IBS patients [94,95]. These similarities make difficult to differentiate between NCGS and IBS cases. However, there are some differences that clinicians and researchers could consider to properly distinguish between NCGS and IBS cases (Table 4). Although the gastrointestinal symptoms triggered in NCGS and IBS cases commonly overlap, NCGS patients trigger extraintestinal symptoms more frequently than IBS ones [96]. Furthermore, although wheat components can trigger symptoms in IBS patients, other foods components other than gluten-containing cereals can also trigger symptoms in IBS patients while the symptoms triggered in NCGS cases are restricted to components found in gluten-containing cereals components. Recently, a diagnostic algorithm to distinguish NCGS from diarrhoea-predominant IBS (IBS-D) was developed. Gender, zonulin serum levels and abdominal symptoms could be used to distinguish NCGS from IBS-D cases, with high accuracy, specificity, and sensitivity values (89.0%, 79.1%, and 90.6%, respectively). The index for discriminating(NvI) developed in this study is interpreted as follows: (1) values <1 can be considered to be IBS-D cases, and (2) values >1 can be considered NCGS cases [84]. Although more research is needed to validate these findings, this approach can be helpful to clinicians and researchers in the differential diagnosis work-up of NCGS and IBS-D cases.

## 7. Dietary Treatment

A GFD is the treatment of choice in NCGS cases, although it remains to be elucidated if a life-long GFD is needed as it is in CD cases. A study by Carroccio et al. reported that 74% of NCGS patients were still following a wheat-free diet after 8 years of their diagnosis and that the consumption of wheat could still trigger symptoms [97]. Although a GFD diet can ameliorate the clinical manifestations of NCGS, there are several issues regarding its implementation. A GFD is associated with an increased intake of macronutrients, such as saturated fats, lipids, and sugar, in addition to calories, and with a decreased intake of micronutrients such as iron, folate, zinc, and others [98]. In line with the previous, it has been reported that subjects with self-reported NCGS that are following a GFD show a higher intake of saturated fat and a lower intake of fiber and micronutrients than subjects on a regular diet [99]. Another study reported that patients with diagnosis of NCGS consume lower amounts of fiber, proteins, carbohydrates and polyunsaturated fatty acids in than healthy controls [100]. On the other hand, a GFD is associated with an increased socioeconomic burden due to gluten-free products are generally more expensive than their regular counterparts [101]. Thus, clinicians should clearly assess the contribution of gluten in the development of NCGS. In line with this, data suggest that NCGS patients present different gluten tolerance thresholds [102]. Therefore, clinicians should assess the tolerance levels of NCGS patients to gluten, to determine if a strict GFD is necessary. Some authors had suggested that a gluten rechallenge could be implemented after 1–2 years of following a GFD and then determinate the adequate dose of gluten that the patient can tolerate [76].

**Figure 3:**
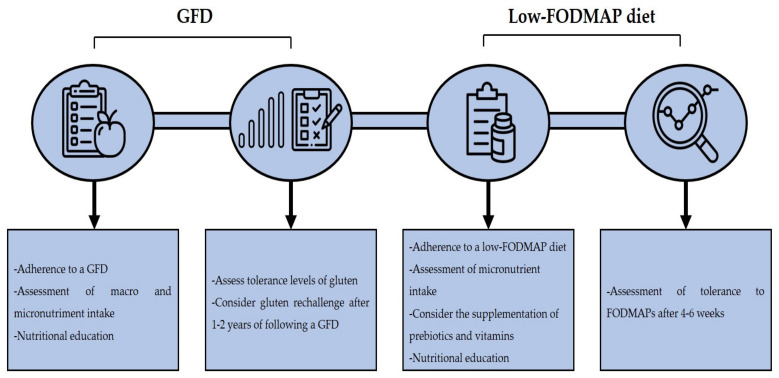
Dietary management in NCGS. Acronyms. GFD: gluten-free diet, FODMAP: fermentable oligo-, di-, monosaccharides, and polyols.

Although a GFD effectively reduces the symptom score after its implementation, some NCGS patients still report symptoms despite being in a strict GFD after years of their diagnosis [103]. A low-FODMAP diet can reduce the symptom score in NCGS patients [74], but the implementation of this diet should be carefully considered as it has been associated with a low intake of natural antioxidants and micronutrients intake. Also, FODMAPs have a prebiotic effect in the colon bacteria, stimulating the growth of *Lactobacilli* and *Bifidobacteria*, and limiting the colonization of *Bacteroides* spp., *Escherichia coli*, and *Clostridium* spp. [33]. An improvement in the lipid metabolism, better absorption of calcium and protective effects against colorectal cancer had also been associated with FODMAP intake [77]. Therefore, supplementation with prebiotics and vitamins is recommended in patients that are following a low-FODMAP diet [104]. Furthermore, a strict follow-up by a trained dietitian is recommended to assess the nutritional intake of the patients. In fact, it has been reported that the nutritional intake of CD patients that are following a GFD and a low-FODMAP diet does not significantly differ in comparison to patients that are only following a GFD when they are supervised by a trained dietitian [105]. A follow-up after 4 to 6 weeks of the implementation of a low-FODMAP diet is recommended in order to assess the patient’s outcome to consider the reintroduction of high-FODMAP foods in the diet [106]. In general, the implementation of a GFD and a low-FODMAP diet in NCGS patients should be considered if improvement of clinical manifestations is seen, but medical and dietitian advice is recommended to prevent any nutritional deficiencies that could appear due to the dietary restrictions (Figure 3).

## 8. Perspectives

Our understanding of NCGS is still at early stages and there are several challenges that clinicians and researchers have to face during the identification of NCGS cases. In the present review, we have given updated information about NCGS epidemiology, pathogenesis, dietary treatment, and biomarkers for its diagnosis. Although there are several proposed biomarkers for the diagnosis of NCGS, all of them lack of sensitivity and specificity [16]. DBPC gluten challenges remain as the gold standard for diagnosing NCGS, but these challenges are difficult to carry out in clinical practice and the appropriate gluten vehicle and placebo remains to be developed. Consequently, due the lack of sensitive and reproducible biomarkers for NCGS diagnosis and an adequate diagnosis approach to be used in clinical practice, the real prevalence of NCGS remains unknown and current evidence of its prevalence is based on survey studies. Similarly, the pathogenesis of NCGS remains to be elucidated although current evidence suggests an involvement of the innate and adaptive immune systems. Researchers should make efforts to elucidate the specific role of the dietary triggers of NCGS and their interaction with the immune system. Finally, dietary counseling by a health professional should always be encouraged since the GFD or a low-FODMAP diet can lead to nutritional imbalance.

## Figures and Tables

**Table 1:** Prevalence of NCGS across survey studies.

Study—Country [Reference]	Settings	Approach	Criteria to Define NCGS	Number of Participants	Outcome (Prevalence)
Aziz et al., 2013—United Kingdom [31]	At population level	Self-administered questionnaire	(1) Absence of SRPD of CD, (2) NCGS-related symptoms	1002	13%
Volta et al., 2014—Italy [23]	Patients attending to clinical centers	Questionnaire and objective diagnostic tests	(1) Patients with suspected NCGS, (2) Exclusion of CD and WA by analytical tests, (3) Clinical assessmens while the patients were on a GFD and after a gluten challenge	12,255	3.19%
Ontiveros et al., 2015—México [25]	At population level	Self-administered questionnaire	(1) Adverse reactions to wheat/gluten, (2) Absence of SRPD of CD and WA, (3) Negative self-reported WA, 4) Adherence to a GFD	1238	0.97%
Cabrera-Chávez et al., 2016—Colombia [26]	At population level	Self-administered questionnaire	(1) Adverse reactions to wheat/gluten, (2) Absence of SRPD of CD and WA, (3) Negative self-reported WA, (4) Adherence to a GFD	1207	4.50%
Cabrera-Chávez et al., 2017—Argentina [27]	At population level	Self-administered questionnaire	(1) Adverse reactions to wheat/gluten, (2) Absence of SRPD of CD and WA, (3) Negative self-reported WA, (4) Adherence to a GFD	1209	6.28%
Carroccio et al., 2017—Italy [22]	High-school students	Self-administered questionnaire	(1) Adverse reactions to wheat at least once per week	555	12.20%
Potter et al., 2018—Australia [20]	Participants follow-up from a previous survey	Self-administered questionnaire	(1) Adverse reactions to wheat-based foods, (2) Absence of SRPD of CD, IBS, and colon cancer	3542	14.90%
Ontiveros et al., 2018—El Salvador [28]	At population level	Self-administered questionnaire	(1) Adverse reactions to wheat/gluten, (2) Absence of SRPD of CD and WA, (3) Negative self-reported WA, (4) Adherence to a GFD	1326	0.98%
Potter et al., 2020—Australia [21]	Participants follow-up from a previous survey	Self-administered questionnaire	(1) Adverse reactions to wheat-based foods, (2) Absence of SRPD of CD, IBS, and colon cancer	1322	13.90%
Arámburo-Gálvez et al., 2020—Brazil [29]	At population level	Self-administered questionnaire	(1) Adverse reactions to wheat/gluten, (2) Absence of SRPD of CD and WA, (3) Negative self-reported WA	1654	1.71%
Araya et al., 2020—Chile [24]	At population level	Self-administered questionnaire	(1) Adverse reactions to wheat/gluten, (2) Absence of SRPD of CD and WA, (3) Negative self-reported WA	1203	0.49%
Ontiveros et al., 2021—Paraguay [30]	Online survey	Self-administered questionnaire	(1) Adverse reactions to wheat/gluten, (2) Absence of SRPD of CD and WA, (3) Negative self-reported WA	1058	5.19%

Acronyms. NCGS: non-celiac gluten sensitivity, CD: celiac disease, WA: wheat allergy, SRPD: self-reported physician diagnosed, GFD: gluten-free diet.

**Table 2:** Potential biomarkers for the diagnosis of NCGS.

Study	Patients	Sample/Assay	Potential Biomarkers Reported	Main Outcomes	Conclusions	Study Limitations
Losurdo et al., 2017 [55]	(1) 20 NCGS (2) 16 CD (3) 16 HS	Sample: Duodenal and rectal biopsies Assay: Immunohistochemistry	- CD4 (T helper lymphocytes) - CD117 (Mast cells) - CD3 (intraepithelial lymphocytes)	CD4: NCGS patients show lower levels of CD4 cells (31.0 ± 22.2 cells/mm^2^) than CD patients (103.7 ± 15.7 cells/mm^2^, sensitivity of 100% and specificity of 90%), and HC (72.5 ± 29.5 cells/mm^2^, sensitivity of 87.5% and specificity of 85%) CD117: NCGS patients showed higher levels of CD117 cells (145.8 ± 49.9 cells/mm^2^) than CD patients (113.5 ± 23.4 cells/mm^2^, sensitivity of 75% and specificity of 55%), and HC (121.3 ± 13.1 cells/mm^2^, sensitivity of 75% and specificity of 55%). CD3: NCGS patients showed higher levels of CD3 cells (18.5 ± 6.4 cells/100 enterocytes) than HS (11.9 ± 2.8 cells/100 enterocytes), but lower than CD patients (40.8 ± 8.1 cells/100 enterocytes). Sensitivity and specificity of <50% for both assessments.	The characterization of CD4, CD117, and CD3 levels could be useful for the clinical diagnosis of NCGS.	Small sample size
Zanini et al., 2017 [38]	(1) 18 NCGS (2) 10 Control	Sample: Duodenal and gastric antrum samples Assay: Hematoxylin and eosin staining and immunohistochemistry	Eosinophils and T lymphocytes distribution	Eosinophils: NCGS patients showed higher eosinophil count in the lamina propria (> 5 eosinophils per HPF × 40) in comparison to the control group (≤ 5 eosinophils per HPF × 40). T helper lymphocytes: NCGS patients showed a normal intraepithelial lymphocyte count (>25 IEL/100 epithelial cells), but with a peculiar distribution in the intestinal epithelium (clusters of IELs in the superficial epithelium and in a linear disposition in the deeper part of the mucosa)	The increased eosinophil count in the lamina propria and the peculiar distribution of IELs may be useful to identify patients with NCGS	- Small sample size - Absence of a complete match with the DBPC challenge.
Carroccio et al., 2018 [37]	(1) 78 NCGS (2) 39 Non-NCGS (3) 16 CD	Sample: Duodenal samples Assay: Immunohistochemistry	Intraepithelial CD3^+^ T cells and eosinophils	Eosinophil infiltration was higher in the rectum and duodenum of NCGS participants than in to non-NCGS participants (*p* < 0.0001). A significant difference between NCGS patients and CD ones was not observed (*p* > 0.05) Intraepithelial CD3^+^ T cells were than in the duodenum of NCGS participants in comparison to non-NCGS participants (*p* < 0.03), but lower than CD patients (*p* < 0.001).	It seems that inflammation of the whole intestine is involved in the pathogenesis of NCGS. Eosinophils appear to be a promising biomarker for NCGS diagnosis.	- Possible selection bias - Lack of asymptomatic controls
Clemente et al., 2019 [83]	(1) 40 NCGS (2) 42 controls with wheat symptoms (3) 24 CD	Sample: Duodenal biopsies and peripheral blood leukocytes Assay: miScript miRNA PCR arrays and Q-PCR	6 miRNAs	Discriminant analysis predicts that the assessment of these 6 miRNAs have a classification accuracy of 60% in CD patients and 81.5% in NCGS patients. Further analysis by PC shows that PC1 correlates with the presence of NCGS (75%), and a ROC curve indicates that PC1 values show a 76% probability to identify NCGS patients.	miRNA signatures may be useful in the diagnosis of NCGS patients	Did not perform a DBPC to confirm the diagnosis of NCGS.
Barbaro et al., 2020 [84]	(1) 86 NCGS (2) 59 IBS-D (3) 25 AC	Sample: Blood samples Assay: ELISA	Serum Zonulin	Once CD is properly rule out, the evaluation of a specific set of variables (female sex, zonulin serum levels, and abdominal pain) allows to differentiate NCGS patients from IBS-D ones with high accuracy (89.0%), specificity (79.1%), and sensitivity values (90.6%).	Zonulin assessment could be used as a diagnostic biomarker of NCGS.	Controverse about the effectiveness of the ELISA kit used [85]
Efthymakis et al., 2020 [49]	(1) 12 NCGS (2) 7 Controls	Sample: Duodenal biopsies Assay: Microarray analysis	15 RNA transcripts	A penalized logistic regression using the LASSO method identify 15 transcripts that mainly contribute to characterize NCGS patients from controls. A ROC curve developed with transcripts showed that one transcript would be sufficient to categorize NCGS patients with high confidence.	The gene expression profile of the intestinal mucosa might be a useful assessment to diagnose NCGS patients.	- Did not perform a DBPC to confirm the diagnose of NCGS. - Lack of a external cohort - Small sample size
Masaebi et al., 2020 [81]	(1) 15 NCGS (2) 110 CD (3) 46 HS	Sample: Peripheral blood Assay: ELISA	IL-15 IL-8	IL-15 showed the highest sensitivity (82.70%), specificity (56.50%), positive predictive value (81.98%), and negative predictive value (57.78%) to differentiate NCGS patients from CD ones, followed by IL-8 (sensitivity: 74.50%, specificity: 73.30%, positive predictive value: 95.35%, and negative predictive value: 30.21%)	The characterization of IL-15 and IL-8 may be useful to differentiate CD patients from NCGS patients and healthy controls.	- Small sample size - Did not perform a DBPC to confirm the diagnosis of NCGS.

Acronyms: NCGS: non-celiac gluten sensitivity, CD: celiac disease, WA: wheat allergy, HS: healthy subjects, AC: asymptomatic controls, IBS-D: diarrhea predominant irritable bowel syndrome, IL: interleukine.

**Table 3:** Comparison among gluten-related disorders characteristics.

	NCGS	CD	WA
Trigger	Gluten, ATIs, FODMAPs	Gluten	Wheat proteins
Prevalence	0.49–14.9%	1%	1%
Pathogenesis	Predominant role of the innate immunity	Autoimmune	IgE-mediated allergenic reaction
HLA DQ2/DQ8	50% carry HLA DQ2/DQ8 haplotypes	>95% carry HLA DQ2/DQ8 haplotypes	No HLA DQ2/DQ8 restricted
Serological biomarkers	There is a lack of serological biomarkers (50% cases are IgG AGA positive)	IgA EMA, IgA tTG, IgG DGP	IgE against wheat-proteins
Histology	Marsh 0 to I	Marsh I to IV	Normal
Type of symptoms	Intestinal and extraintestinal	Intestinal and extraintestinal	Intestinal and extraintestinal
Onset of symptoms	Hours to days	Days to weeks	Minutes to hours
Symptoms intensity	Mild	Low to High	Low to High
Complications	Unknown	Long-term complications	Anaphylaxis
Diagnosis	DBPCGC	HLA DQ2/DQ8, antibodies and biopsy	IgE against wheat, skin-prick test and wheat challenge
Treatment	GFD, low-FODMAP diet	GFD	Wheat-free diet
Treatment duration	Unknown	Life-long	Life-long

Acronyms: NCGS: non-celiac gluten sensitivity, CD: celiac disease, WA: wheat allergy, ATIs: amylase and trypsine inhibitors, FODMAPs: fermentable oligo-, di-, monosaccharides, and polyols, AGA: anti-gliadin antibodies, DGP: deaminated gliadin peptides, EMA: endomysial, tTG: tissue transglutaminase, DBPCGC: double-blind placebo-controlled gluten challenge, GFD: gluten-free diet.

**Table 4:** Comparison among NCGS and IBS characteristics.

	NCGS	IBS
Food trigger	Gluten, ATIs, FODMAPs	Not restricted to wheat components found in gluten-containing cereals
Prevalence	0.49–14.9%	1.1–35.5%
HLA DQ2/DQ8	50% presents HLA DQ2/DQ8 haplotypes	No HLA DQ2/DQ8 restricted
Histology	Marsh 0 to I	Normal
Clinical manifestations	Intestinal and extraintestinal	Intestinal
Diagnosis	DBPC gluten challenge	Rome IV criteria
NvI	>1	<1
Treatment	GFD, low-FODMAP diet	Multi-approach treatment (may benefit with a GFD and low-FODMAP diet)

Acronyms. NCGS: non-celiac gluten sensitivity, IBS: Irritable bowel syndrome, FODMAPs: fermentable oligo-, di-, monosaccharides, and polyols, DBPCGC: double-blind placebo-controlled gluten challenge, NvI: index for discriminating, GFD: gluten-free diet.

## Data Availability

Not applicable.
